# SUMO proteins in the cardiovascular system: friend or foe?

**DOI:** 10.1186/s12929-020-00689-0

**Published:** 2020-10-24

**Authors:** Prithviraj Manohar Vijaya Shetty, Ashraf Yusuf Rangrez, Norbert Frey

**Affiliations:** 1 Department of Internal Medicine III (Cardiology, Angiology, Intensive Care), University Medical Center Kiel, Rosalind-Franklin Str. 12, 24105 Kiel, Germany; 2 Manipal Institute of Regenerative Medicine, MAHE-Bengaluru, Bangalore, India; 3 DZHK (German Centre for Cardiovascular Research), Partner Site Hamburg/Kiel/Lübeck, Kiel, Germany

**Keywords:** Post-translational modification, SUMO, SUMOylation, Cardiovascular diseases

## Abstract

Post-translational modifications (PTMs) are crucial for the adaptation of various signalling pathways to ensure cellular homeostasis and proper adaptation to stress. PTM is a covalent addition of a small chemical functional group such as a phosphate group (phosphorylation), methyl group (methylation), or acetyl group (acetylation); lipids like hydrophobic isoprene polymers (isoprenylation); sugars such as a glycosyl group (glycosylation); or even small peptides such as ubiquitin (ubiquitination), SUMO (SUMOylation), NEDD8 (neddylation), etc. SUMO modification changes the function and/or fate of the protein especially under stress conditions, and the consequences of this conjugation can be appreciated from development to diverse disease processes. The impact of SUMOylation in disease has not been monotonous, rather SUMO is found playing a role on both sides of the coin either facilitating or impeding disease progression. Several recent studies have implicated SUMO proteins as key regulators in various cardiovascular disorders. The focus of this review is thus to summarize the current knowledge on the role of the SUMO family in the pathophysiology of cardiovascular diseases.

## Introduction

Every cell has countless biological processes occurring simultaneously, right from its genesis till its terminus, recalibrating its fate perpetually. Each of these processes such as growth, molecule and ion transport or cell division involves molecular pathways with an array of intermediates which ultimately converge on a gene or a group of genes getting modulated, which further regulate a cascade of more such pathways. Although the effect of each pathway is fairly pre-determined, a small molecular modification in one of the players can shift the entire direction of a signaling cascade, altering the fate of the intermediates and definitely changing the outcome. Such modifications, typically post-translational modifications (PTM), can serve as a ‘by-default setting’ in the pathway or could be brought about by the state at which the cell is at that instant, e.g. in a situation of cellular stress.

Small Ubiquitin like Modifier (SUMO) proteins have been recognized as one of the key PTMs modulating the function and half-life of many proteins, thereby serving as master switches in multiple molecular signalling pathways. In the following, we provide a detailed overview on the individual SUMO proteins and their (patho)physiological roles.

### The SUMO family

SUMO is a conserved family of proteins that has one member in yeast, around eight in plants and four in mammals. In the late 90s, a single protein, now called SUMO1, was reported by several researchers with different names with differing functions. SUMO1 was found to be associated with DNA recombination and DNA damage response proteins RAD 51/52, and since it showed similarity to ubiquitin, it was termed as ubiquitin-like protein 1 (UBL1) [101]; in another report it prevented cell death by associating with the cytoplasmic tails or the “*death domains*” of FAS/APO 1 and tumour necrosis factor (TNF) receptor 1 and thus was given the name Sentrin (after ‘*sentry’* for the protein’s function as a guardian) [85]; moreover, due to interaction with promyelocytic leukaemia (PML) protein,, it was also named as PIC1 (PML interacting clone 1) [10]; its posttranslational modification of RanGAP1 with subsequent translocation to RanBP2, a nuclear pore complex protein, led to the name GAP modifying protein 1 (GMP1) or SUMO1 [72, 74]; whereas, homology with the yeast protein Smt3 gave SUMO1 the name Smt3a [62]. To the contrary, SUMO2 and SUMO3 (SUMO2/3) were discovered using a database search [51]. SUMO2/3 share almost 95% homology with one other, but the two share only around 50% homology with SUMO1. SUMO4 was identified while studying the association of single nucleotide polymorphisms with type I diabetes, however, it is currently viewed as an intron-less pseudogene [12, 30]. The latest member of the family, a primate-specific SUMO5 with high tissue specificity, was found to facilitate promyelocytic leukaemia nuclear body growth [67].

Despite the similarity of SUMO with ubiquitin in sequence, size and even conjugation, their functions differ drastically. Ubiquitin is best known for its role in redirecting the proteins towards degradation via proteasomal or lysosomal pathways by binding to the target protein in a polymer fashion (also known as poly-ubiquitin tags), but it is also involved in regulating nuclear localization and DNA repair pathways [65]. SUMO, on the other hand, has been reported to play a role in a plethora of functions like DNA replication, transcriptional regulation, chromatin organization, cell cycle regulation, sub-cellular localization, protein–protein interactions, protein–DNA interactions, DNA damage response, and degradation [27, 33, 70, 74, 90, 100, 105, 108]. It is interesting and important to note that either ubiquitination or SUMOylation of a single substrate results in a different outcome. For example, ubiquitination of Histone deacetylase 1 (HDAC1), an epigenetic regulator, sends the protein to proteasome mediated degradation, whereas, SUMOylation promotes transcriptional repression of HDAC1 by enhancing its histone deacetylation activity [16]. The so called ‘*Guardian of the genome*’, p53 and the tumour suppressor promyelocytic leukaemia (PML) protein both get degraded upon ubiquitination. However, SUMOylation of PML is a prerequisite for nuclear body formation [124] and SUMO conjugation relegates the transcriptional activity of p53 [116].

### The SUMO cycle

**Fig. 1:**
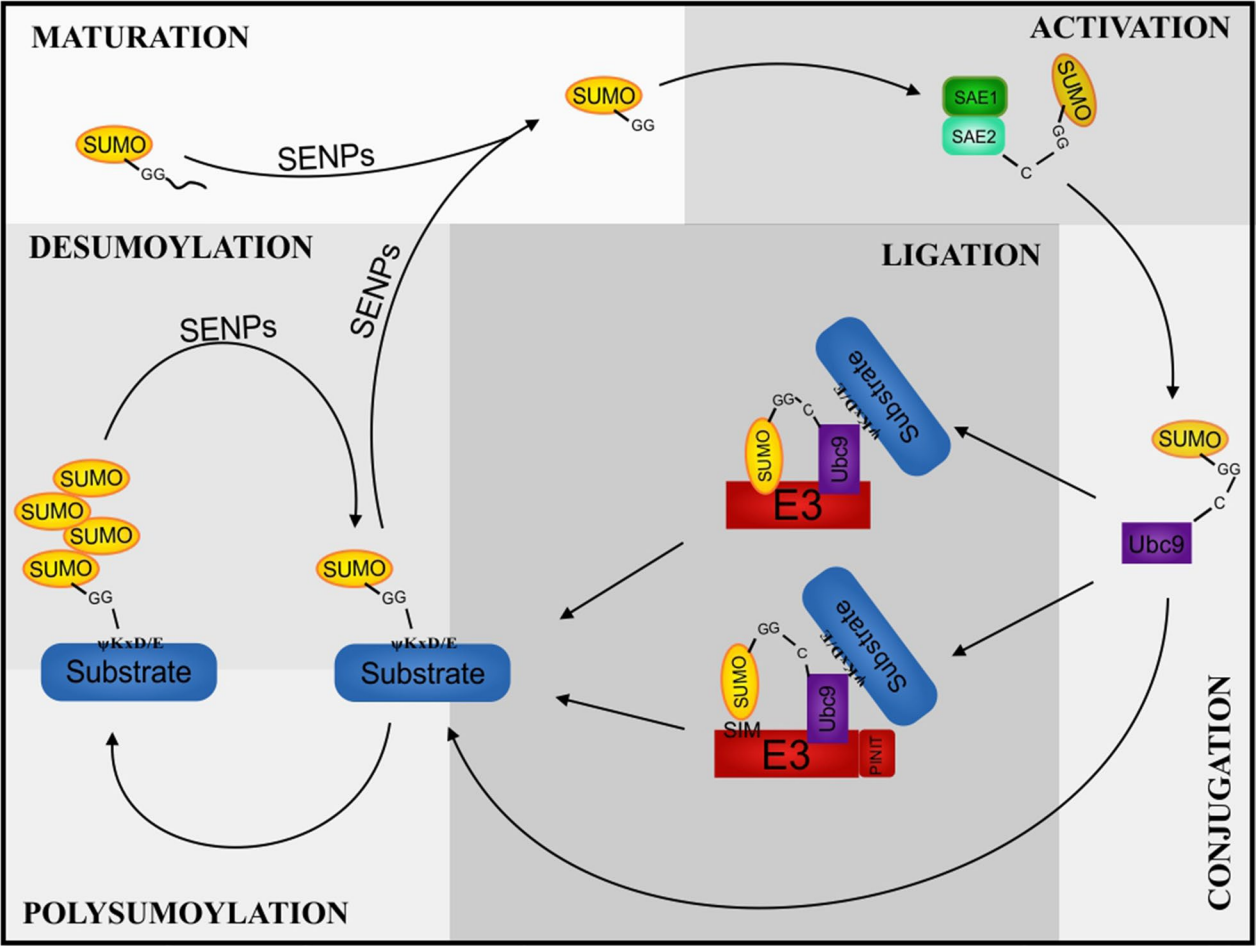
The SUMO cycle. SUMO is produced as a precursor which is processed by SENP to expose the C terminal diglycine residues (MATURATION). SUMO is then activated by heterodimer E1 enzyme in an ATP dependent manner (ACTIVATION). SUMO is then transferred to the SUMO E2 conjugating enzyme, Ubc9 (CONJUGATION). Further, SUMO is attached to the substrate either in the presence of E3 (with or without substrate binding) or directly by Ubc9 (LIGATION). Depending on the substrate and function, more than one SUMO molecules can be bound (POLYSUMOYLATION). SUMOylation is reversed by SENPs, releasing the SUMO molecule(s) for the next cycle

SUMO proteins are synthesized as propeptides and the maturation of these proteins requires the activity of proteins called the SENPs (sentrin/SUMO-specific proteases). SENPs chop off four amino acid residues from the SUMO propeptide, exposing the diglycine residue, thereby rendering the protein ready for substrate conjugation. The SUMO conjugation pathway is very similar to the ubiquitin conjugation pathway (Fig. 1). Post maturation, the SUMO molecule moves to the activation step by an E1 activating enzyme. In mammals, a SAE1–SAE2 heterodimer catalyses the adenylation of SUMO in the presence of Mg^2+^ ions [20, 84, 98]. This high energy bond between AMP and SUMO is broken and a new thioester linkage is formed between the active cysteine residue of SAE2 and the C terminal glycine of SUMO [49]. The next step is conjugation where SUMO is transferred to Ubc9, a SUMO conjugating E2 enzyme. The association between SUMO and the active cysteine of Ubc9 is another thioester bond [26, 48, 63, 99]. The penultimate step of the cycle is SUMO conjugation to a substrate. Ubc9 is potent enough to execute the conjugation process, but there have also been studies that suggest the involvement of E3 ligases in this process. Unlike ubiquitin E3 ligases, SUMO E3s act as a scaffold bringing the substrate and the SUMO bound E2 in proper orientation for the conjugation to materialize. SUMO E3 ligases have been classified into different families. PIAS (protein inhibitor of activated STAT) is a eukaryotic SUMO E3 ligase family with its counterparts called Siz proteins in the yeast. PIAS have two domains, one called the SAP domain for binding to AT rich DNA sequences while the other called the Siz/PIAS (SP) RING domain like the ubiquitin E3s, which allow for attachment with E2 [39]. PIAS also has the capability of binding to the substrate covalently via a PINIT domain [87]. However, its attachment to the SUMO molecule is non-covalent via a SUMO interacting motif (SIM) present on PIAS [75]. Ran binding protein 2 (RanBP2) is another SUMO E3 ligase family containing an internal repeat (IR) domain which facilitates the ligase activity. This family of E3s bind to Ubc9 promoting an optimal orientation but interestingly does not bind to the substrate [81, 97]. Polycomb 2 (Pc2) is a multimeric complex SUMO E3 ligase which does not possess the RING domain, yet binds to both Ubc9 and the substrate to promote the conjugation [50]. ZNF451 is the latest family added to the SUMO E3 ligase list. ZNF451 proteins have tandem SIM and inter SIM motifs which help perform the SUMO ligation [52]. Along with their ligation (E3) function, ZNF451 proteins also indulge in the SUMO elongation (E4) function [23]. Extensive research has revealed that SUMO substrates have a consensus sequence where the SUMO proteins bind. Interestingly, this sequence is also present in SUMO2/3 but not in SUMO1. This could be the reason why the former form polymeric chains while the latter does not [60]. In fact, this also corresponds to the presence of large pools of free SUMO2/3 but comparatively little SUMO1 [96]. Like deubiquitylation, the SUMO cycle concludes with deconjugation of SUMO by SENPs, setting the SUMO proteins free to enter the cycle again for changing the fate of another molecule (Fig. 1).

### SUMO consensus motif v/s SUMO interacting motifs

Consensus motifs for SUMO conjugation are specific sequences in the SUMO substrates which are recognized by the E2 enzymes. This consensus motif is a highly conserved 4 amino acid sequence ψKxD/E, wherein ψ stands for a large hydrophobic amino acid and x is any amino acid [95]. Substrate recognition is assisted by the electrostatic interaction and the hydrogen bonds between E2 and the residues surrounding the lysine of this motif. A spike in the rate of reaction is brought about by the decrease in the pKa of the lysine residue via the residues in the E2, and further stabilization of this interaction is facilitated by co-factors and E3 enzymes. For example, SUMOylation of Peroxisome proliferator-activated receptor (PPAR)-γ2 at k107 is enhanced by the phosphorylation at S112; such motifs with a phosphorylatable serine residue at the C-terminal of the consensus motif, ψKX(D/E)XXSp, are called phosphorylation-dependent SUMO motifs (PDSMs) [119]. This phosphate group interacts with the basic patch in the Ubc9, aiding in recognition [76]. Another example of such a variant are negatively charged amino acid-dependent SUMO motifs (NDSMs), also present at the C-terminus of the consensus and important for the recognition of some proteins by Ubc9 [76, 120].

Around half of the SUMO substrates bind via the consensus motif, yet upon cellular stress conditions, interactions with non-consensus motifs seem to surge. In the early 2000s, a group of investigators noticed a few proteins interacting with an already SUMOylated p73, a tumour suppressor. Upon further investigation, they found that a SxS sequence (S = Serine) between a hydrophobic stretch and an acidic stretch in the protein is necessary for this interaction [75]. Further on, a few other groups showed that a (V/I)X(V/I)(V/I) motif, a hydrophobic core in between acidic stretches, is the obligatory sequence in this interaction [103]. Such sequences are called SIMs, another set of loose consensus motif bringing about a non-covalent interaction between the protein and a SUMO molecule. For this interaction, the beta sheets in the SIMs take on a parallel or an antiparallel orientation with the beta sheet of the SUMO molecule, where, the acidic residues around the SIMs and the SUMO basic residues form salt bridges [25, 53]. Like the PDSMs, SIMs also have a family which comprises of a phosphorylatable serine residue. Phosphorylation of this serine residue at the C terminal of the hydrophobic core by casein kinase 2 augments the SUMO-substrate interaction [22].

## SUMOylation in cardiovascular diseases

### Atherosclerosis

**Fig. 2:**
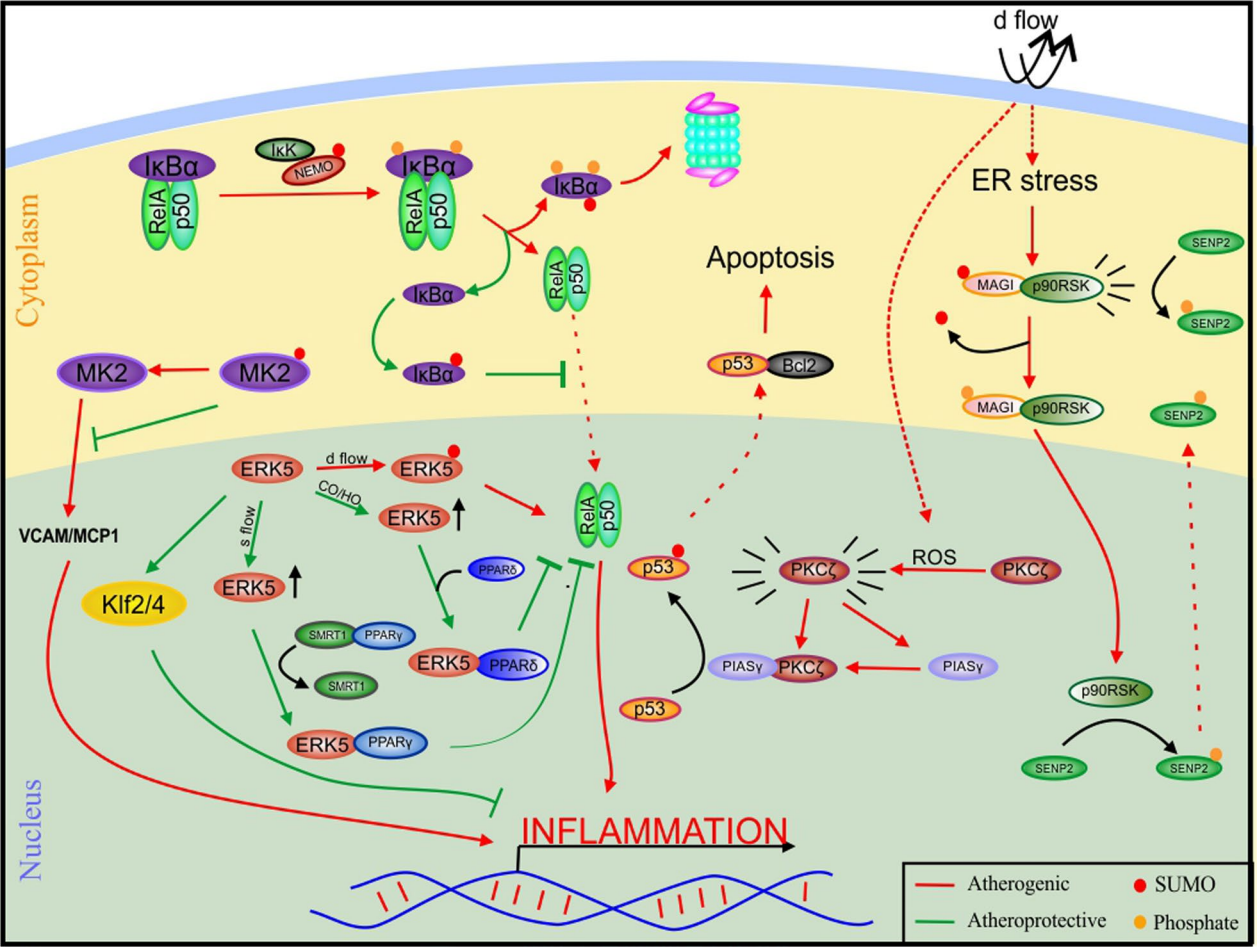
Participation of SUMOylation in molecular signalling pathways involved in atherosclerosis. In the NFκB pathway, SUMOylation of IκBα via SUMO1 proves atheroprotective while IκBα SUMO2/3 conjugation proves atherogenic and so does NEMO SUMOylation. MK2 SUMOylation inhibits VCAM1/MCP1 mediated inflammation acting as a friend while p53 SUMOylation is apoptotic, hence SUMOylation acts as a foe here. SUMO when bound to MAGI activates p30RSK which further stabilizes ERK–SUMO interaction by retaining SENP2 in the cytoplasm, hence SUMOylation here in both the scenarios prove to be hazardous to the cells. *Bcl2* B cell lymphoma 2, *CO* carbon monoxide, *d flow* distributed flow, *ERK5* extracellular-signal-regulated kinase 5, *HO* hemeoxygenase, *IκBα* inhibitor of κB-α, *IκK* inhibitor of κB kinase, *Klf* Kruppel-like Factor, *MAGI* membrane-associated guanylate kinase with inverted domain structure-1, *MCP1* monocyte chemoattractant protein 1, *MK2* mitogen-activated protein kinase-activated protein kinase 2, *NEMO* NFκB essential modulator, *P90RSK* P90 Ribosomal s6 Kinase, *PIASγ* protein inhibitor of activated STAT γ, *PKCζ* protein kinase C ζ, *PPAR* peroxisome proliferator-activated receptor, *s flow* steady flow, *SENP* sentrin/SUMO-specific proteases, *SMRT1* silencing mediator of retinoic acid and thyroid hormone receptor, *VCAM* vascular cell adhesion protein

Atherosclerosis is an inflammatory disorder characterized by accumulation of lipids and immune cells, termed as plaques, on the intima of blood vessels ultimately triggering conditions like myocardial infarction and stroke. These plaques are a result of chronic activation of molecular signalling pathways in the inner endothelial lining of the blood vessels that mediate inflammation and apoptosis (Fig. 2). Signalling inside these cells depends on the flow dynamics of blood in the specific areas. A steady state “laminar” flow is regarded to be atheroprotective, while “turbulent” flow has been shown to be atherogenic [18]. NFκB is a transcription factor which upon activation enters the nucleus and initiates transcription of a number of genes responsible for stimulating the inflammatory signals in the cell. SUMOylation of IκBα, a component of NFκB inhibitor, via SUMO 1 rescues IκBα from ubiquitin mediated proteasomal degradation, in turn disabling the NFκB pathway [19]. On the contrary, SUMOylation of the same molecule via SUMO2/3 detaches it from NFκB, stimulating this pathway [19]. A component of the NFκB inhibitor kinase, NEMO, acts as a stimulant for NFκB upon SUMOylation [45].

Mitogen-activated protein kinase-activated protein kinase 2 (MAPKAPK2, or MK2) is another proinflammatory molecule which can further surge NFκB activation [47]. *LDLR*^−/−^*/MK2*^−/−^ compound-mutant mice showed a reduction in TNFα induced inflammation via downregulation of VCAM1 and MCP1 expression and thus reduction in plaque formation compared to LDLR^−/−^ mice, confirming the proinflammatory role of MK2 [47]. SUMOylation of MK2 inhibits the kinase activity of MK2 in turn inhibiting its inflammatory ability [14].

Extracellular-signal-regulated kinase 5-ERK5 (a member of the MAPK family) is a master molecule which inhibits atherosclerosis either by interacting with various proteins to inhibit inflammatory pathways or by controlling the expression of various anti-atherogenic targets [35]. The steady state flow activates ERK5, which in turn associates with peroxisome proliferator-activated receptor γ (PPARγ) [2]. Binding of ERK5 with the PPARγ-SMRT complex discharges SMRT (Silencing mediator of retinoic acid and thyroid hormone receptor) activating PPARγ for NFκB inhibition [2]. Carbon monoxide and heme oxygenase-1 mediated ERK5 activation also leads to association of ERK5 with PPARδ, rendering it active for NFκB inhibition [115]. ERK5 activation upregulates KLF2 and KLF4 which also help reduce inflammation [80, 83, 89]. SUMOylation of ERK5 occurs due to turbulent flow mediated ROS production, and impedes the atheroprotective role of ERK5 [37]. ROS mediated Protein kinase C ζ (PKCζ) activation brings about the association of activated PKCζ with protein inhibitor of activated STAT protein gamma (PIASγ)- an E3 SUMO ligase [37]. Once the E3 ligase is activated, it SUMOylates p53 resulting in its nuclear expulsion [37]. p53 associates with Bcl2 and inhibits its anti-apoptotic activity, thus activating the apoptotic signal [34]. Upon ER stress caused by distributed flow, the P90 Ribosomal s6 Kinase (p90RSK) and SUMOylated Membrane-associated guanylate kinase with inverted domain structure-1 (MAGI1) complex leads to phosphorylation of both MAGI1 and SENP2 [1, 36]. The phosphorylation of SENP2 enables its cytoplasmic retention, whereas, the phosphorylation of MAGI1 enables its deSUMOylation by SENP1 [1, 36]. The deSUMOylated MAGI1 facilitates the nuclear localization of p90RSK-MAGI1 and ER stress by product ATF6-MAGI1 complex. p30RSK further stabilizes the ERK5-SUMO in the nucleus by phosphorylating the nuclear SENP2 causing its export [1, 36]. Activating transcription factor 3 (ATF3) is a stress response transcription factor downstream of various environmental stresses. In Angiotensin II induced hypertensive mice, the colocalization of ATF3 and SUMO1 was observed in the thoracic aorta [122]. SUMOylation of ATF3 inhibits its ubiquitin mediated degradation and stabilizes the protein. SUMO deficient ATF3 K42R mutant on the other hand showed reduced nitric oxide production and knockdown of ATF3 or SUMO1 inhibited various inflammatory molecules [122]. These results indicate that the SUMOylation acts as a regulatory step in ATF3 mediated endothelial inflammation [122].

Reverse cholesterol transport (RCT) is a mechanism in which cholesterol from the peripheral tissues is delivered back to the liver [31]. A cross between an atherosclerosis-prone LDL receptor knockout mice (*LDLR*^−/−^) with a nuclear receptor liver receptor homolog 1 (LRH1) SUMOylation mutant LRH1 K289R, showed increased RCT in the mice [106]. The reason being, the wild type SUMO binds to prospero homeobox protein 1 (PROX1) and represses hepatic RCT genes, however, the SUMOylation deficient LRH1 fails to interact with PROX1, derepressing the RCT genes and reducing atherosclerosis in the mice [106].

### Cardiomyopathy and heart failure

#### Cardiac hypertrophy

Cardiac hypertrophy is a condition in which cardiomyocytes grow and undergo cytoskeletal remodelling, thus increasing its size and sarcomere stability as a compensatory response to the increased biomechanical stress. However, sustained cardiac hypertrophy is usually maladaptive and results in contractile dysfunction and arrhythmias, clinically associated with heart failure and sudden death. Hypertrophy caused by pressure overload due to transverse aortic constriction is inhibited by SUMO1 gene transfer and left ventricular function is restored [107]. Likewise, oxidative stress mediated modifications in SERCA2a are inhibited by SUMO1 preventing the cardiomyocytes from undergoing hypertrophy [107]. Angiotensin II and insulin growth factor II receptor (IGF-IIR) have also been reported to be involved in cardiac hypertrophy [42]. Heat shock protein-2 (HSP2) SUMOylated at K82 by SUMO1 is unable to bind to the IGF-IIR promoter. A study revealed that angiotensin II upon binding to its receptor upregulates a polycomb protein MEL18, which then mediates deSUMOylation of HSP2 protein allowing it to activate IGF-IIR gene by direct interaction [42]. The increased protein expression of IGF-IIR subsequently leads to cardiac hypertrophy [42]. The possible mechanisms underlying MEL18 mediated deSUMOylation could be either interaction of MEL18 with UBC9 leading to its inhibition or MEL18 activating SENP1 rendering deSUMOylation, or both [64].

**Fig. 3:**
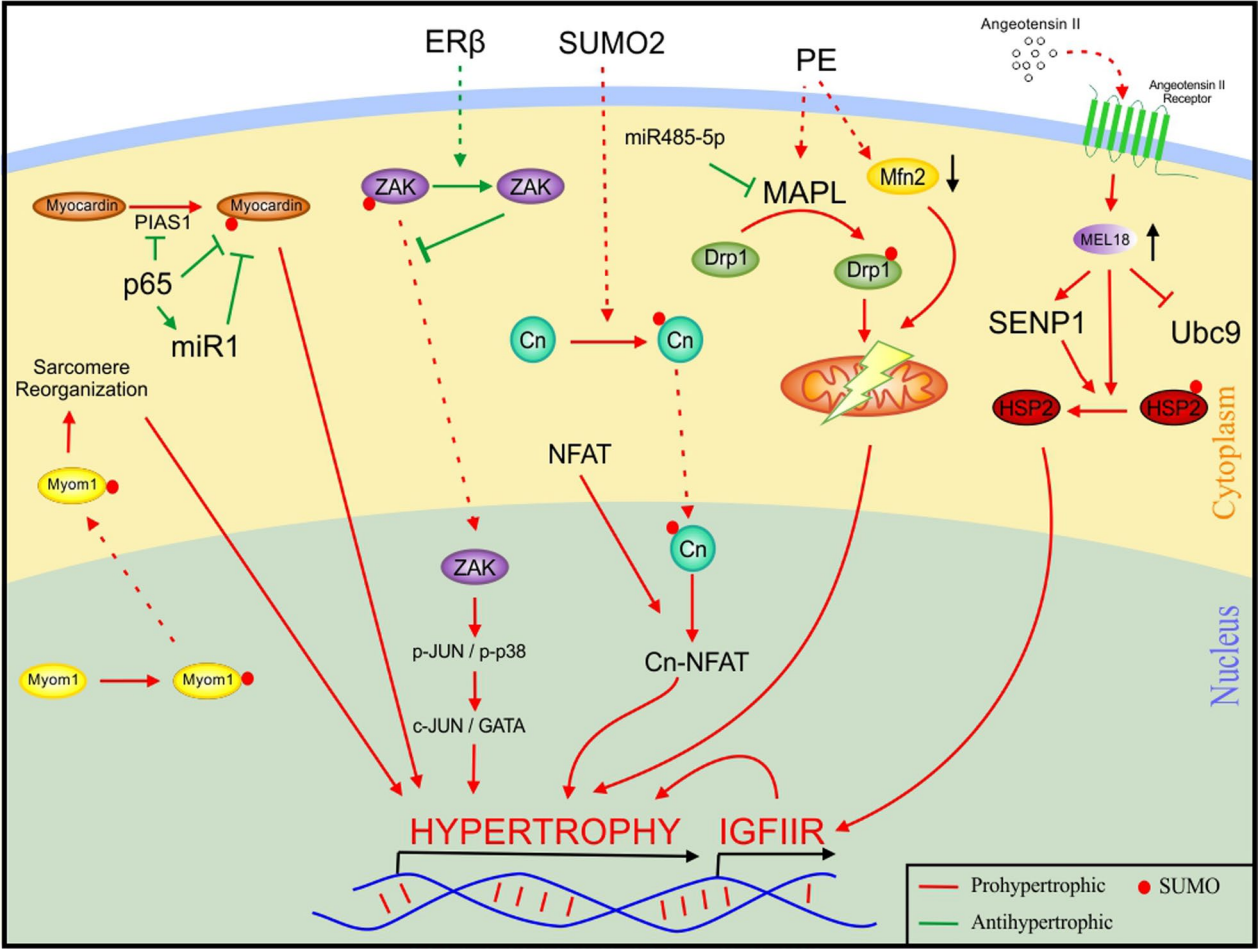
Participation of SUMOylation in molecular signalling pathways involved in cardiac hypertrophy. SUMO conjugation of Cn facilitated NFAT mediated hypertrophy, while SUMO binding to Myocardin could be a direct hypertrophic stimulus. Likewise, SUMOylated Myom1 can cause sarcomeric reorganization and SUMOylated Drp1 can cause mitochondrial disfunction, both leading to cardiac hypertrophy. These portray SUMO as a foe. But, ZAK, which mediates hypertrophy via c-JUN/GATA and HSP, which causes IGFIIR mediated hypertrophy, both upon SUMO conjugation obstructs their hypertrophic role, attesting SUMO as a friend here. *Cn* calcineurin, *Drp1* dynamin related protein 1, *ERβ* oestrogen receptor β, *HSP2* heat shock protein 2, *IGFIIR* Insulin growth factor 2 receptor, *MAPL* mitochondrial anchored protein ligase, *Mfn2* mitochondrial fusion protein2, *Myom1* myomesin 1, *NFAT* nuclear factor of activated T cells, *PE* phenylephrine, *PIAS1* protein inhibitor of activated STAT; *SENP1* sentrin/SUMO-specific proteases, *SUMO2* small ubiquitin related modifier, *ZAK* sterile alpha motif and leucine zipper containing kinase

SUMO1 conjugation of myocardin (a smooth muscle and cardiac specific transcriptional coactivator) assisted by PIAS1 can cause hypertrophy in the heart, which is attenuated by p65 (a unit of NFκB heterodimer), as it inhibits both PIAS1 and SUMO1 [68]. p65 also increases the expression of microRNA miR-1, which in turn silences myocardin [44]. Activation of ZAK (Sterile alpha motif and leucine zipper containing kinase), a pro apoptotic protein from MAPKKK, activates MAPKs like p-JUN and P-P38, which further promotes c-JUN and GATA -induced hypertrophy [40]. ZAK translocate to the nucleus via SUMO1 conjugation, and this is attenuated by oestrogen receptor β (ERβ) overexpression which retains ZAK in the cytoplasm [86]. Overexpression of human myofibrillogenesis regulator 1 (MR1) in NRVCMs showed increased sarcomere organization followed by hypertrophy [113]. MR1 overexpression also showed an increased nuclear to cytoplasmic translocation of myomesin-1 mimicking SUMO1 overexpression [113]. Interestingly, SUMO1 overexpression along with a stealth siRNA for MR1 did not result in nuclear translocation of myomesin-1. These results indicate that MR1 SUMOylates myomesin-1, which then mediates increased sarcomere organization preceding hypertrophy [113]. In 2017, a group of investigators observed that treatment of primary cardiomyocytes with phenylephrine (PE), a hypertrophy causative agent, led to an increase in the expression of the SUMO E3 ligase Mitochondrial anchored protein ligase (MAPL), localized to the mitochondrial membrane [123]. This was accompanied by increased mitochondrial fission and decrease in mitochondrial fusion protein-2 (Mfn2), a protein necessary for mitochondrial fusion [123]. The authors suggested that the hypertrophic stimulus upregulates MAPL which promotes SUMOylation of mitochondrial fission protein Drp1 thus facilitating hypertrophy [123]. They also found a microRNA miR-485-5p to abrogate hypertrophy by silencing MAPL expression [123]. The calcineurin (Cn)-NFAT pathway is one of the most important signalling pathways associated with cardiac hypertrophy [77]. Bernt et al. used a luciferase mediated screening approach to discover SUMO2 as a novel candidate modulator associated with Cn-NFAT signalling [7]. We found that SUMO2 interacts with Cn and translocate Cn to the nucleus envelope whereby it activates NFAT signalling [7]. Overexpression of a mutant diglycine deficient SUMO2 (ΔGG) failed to alter Cn-NFAT signalling, pointing towards a SUMOylation independent activation of the pathway by SUMO2-Cn. These findings summarised in Fig. 3 signify the importance of SUMOylation in cardiac hypertrophy.

#### Dilated cardiomyopathy and heart failure

**Fig. 4:**
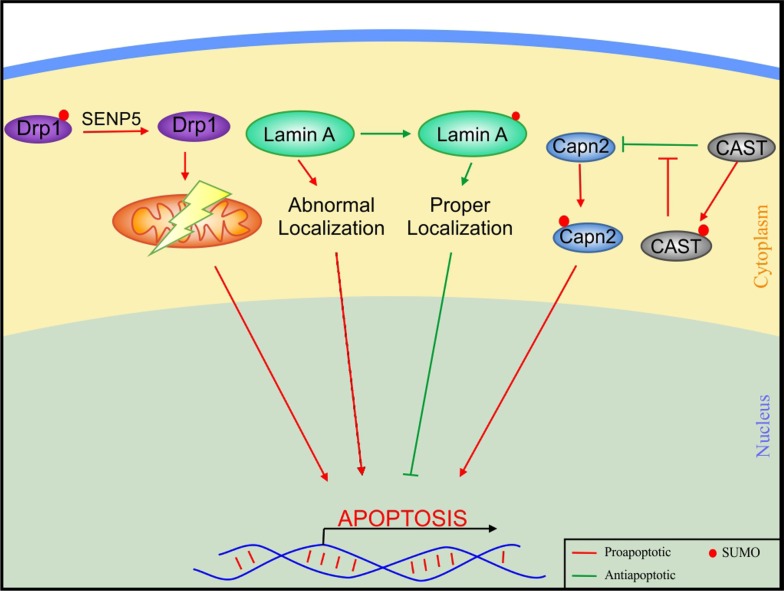
Participation of SUMOylation in molecular signalling pathways involved in dilated cardiomyopathy. SUMO plays a friendly role upon conjugating with Drp1 to inhibit mitochondrial disfunction and binding to Lamin A for its proper localization, but has a harmful effect when binding to CAST and Capn to induce apoptosis. *Capn2* calpain small subunit 2, *CAST* calpastatin, *Drp1* dynamin related protein 1, *SENP* sentrin/SUMO-specific proteases

Dilated cardiomyopathy is a disease characterized by dilation in the left ventricular wall owing to reduction in contractile function. Anomalies in SUMO-modification of several substrates have been shown to contribute to dilated cardiomyopathy (Fig. 4). Cardiac specific overexpression of SENP5 results in mitochondrial dysfunction ultimately leading to dilated cardiomyopathy in aging mice. These adverse effects are caused by SENP5 mediated deSUMOylation of SUMO2/3 modified dynamin related protein (Drp1), a mitochondrial fission protein [58]. Results with SENP2 transgenic mice concurred with that of SENP5 overexpressing mice exhibiting cardiac fibrosis and cardiomyopathy with age [56]. Lamin A, an intermediate filament nuclear envelope protein, reportedly accounts for almost 10% of familial DCM due to autosomal dominant hereditary mutations [32]. SUMOylation of lamin A at K201 in the consensus sequence MKEE is necessary for normal cellular function. Mutants for K201R showed decreased SUMOylation of lamin A and exhibited abnormal lamin A localization and eventual cardiomyopathy, suggesting an important role of lamin A sumoylation in cardiomyopathy [121].

The calpain–calpastatin pathway is an apoptotic pathway with its members found to be associated with SUMO2. Of note, cardiomyopathy is observed in transgenic mice overexpressing SUMO2. The calpain small subunit 2 (Capns2) inhibitor CAST (calpastatin), when conjugated to SUMO2, shows a reduced inhibition ability, whereas SUMO2 conjugation of Capns2 increases the enzymatic activity of Capns2. As a matter of fact, both SUMOylation events here prove to be detrimental in cardiomyopathy and subsequent heart failure [59].

#### Myocardial infarction/ischemic cardiomyopathy

**Fig. 5:**
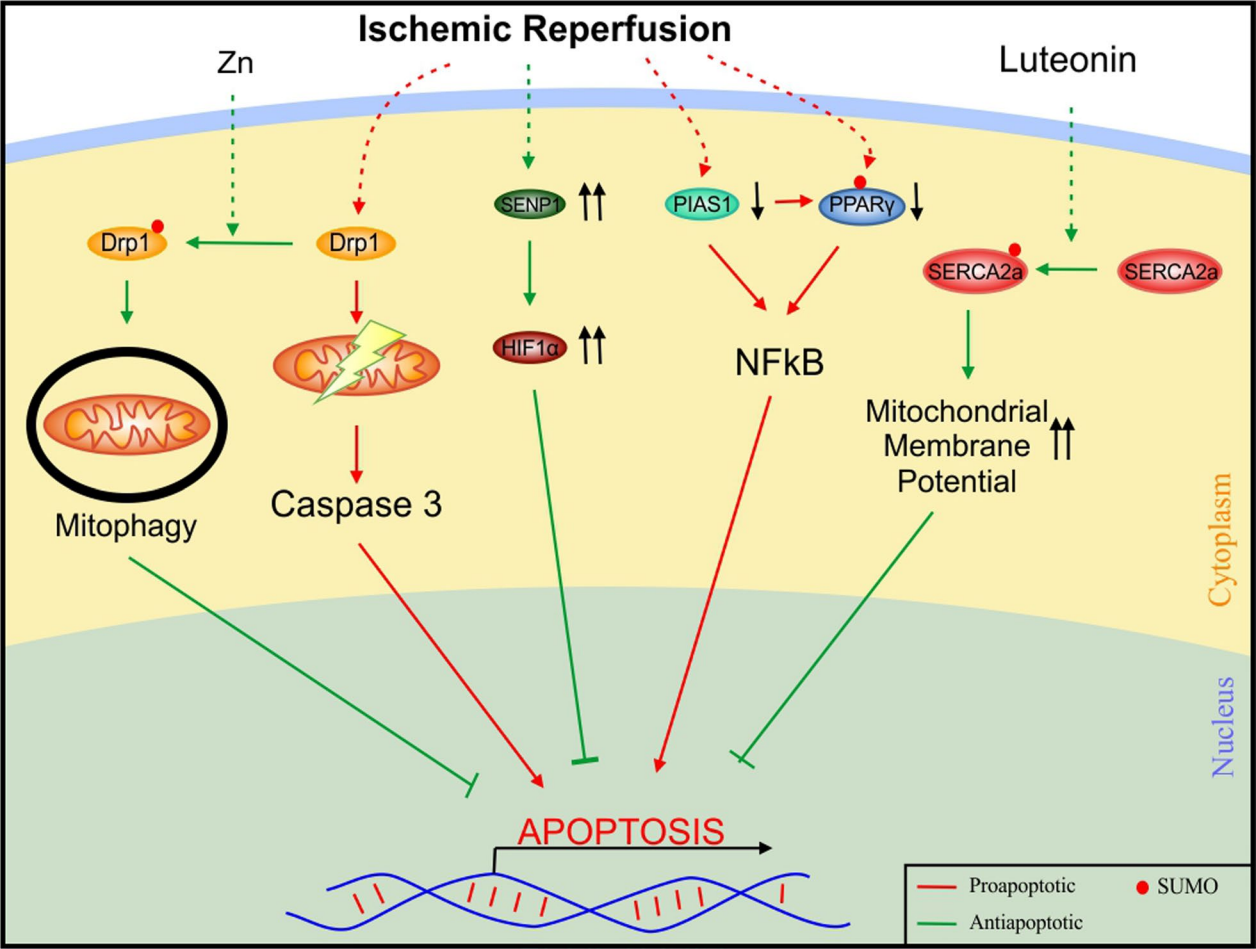
Participation of SUMOylation in molecular signalling pathways involved in ischemic cardiomyopathy. Zinc mediated Drp1 SUMOylation inhibits mitochondrial disfunction and SERCA2a SUMOylation increases mitochondrial membrane potential inhibiting apoptosis demonstrating the friendly nature of SUMO. Decreased levels of SUMOylated PPARγ activates the NFκB pathway, again displaying the beneficial role of SUMO conjugation. *Drp1* dynamin related protein 1, *HIF 1α* hypoxia inducible factor 1α, *NFκB* nuclear factor κB, *PIAS1* protein inhibitor of activated STAT 1, *PPAR* peroxisome proliferator-activated receptor, *SENP* sentrin/SUMO-specific proteases, *SERCA2a* Sarco/endoplasmic reticulum Ca^2+^-ATPase 2a, *Zn* zinc

Ischemia reperfusion injury is the damage caused by the reoxygenation of tissue after a short period of ischemia. Ischemic reperfusion leads to a decrease in the overall SUMO conjugation in cardiomyocytes. Like other forms of cardiomyopathies, SUMOylation defects in certain SUMO substrates causes ischemic cardiomyopathy (Fig. 5). Upon treatment with zinc, SUMO1 mediated SUMOylation of Drp1 increases zinc induced mitophagy protecting the cells against injury [9]. Ischemic reperfusion also increases SENP1 levels which further activates the hypoxia inducible factor 1α (HIF1α) pathway, thus supporting its cardioprotective role [29]. Moreover, there is a decrease in protein inhibitor of activated STAT 1 (PIAS 1) levels, a SUMO E3 ligase, upon ischemia reperfusion. An increased SUMOylation of PPARγ was observed upon ectopic expression of PIAS 1, inhibiting the NFκB pathway. It also revealed a decrease in the phosphorylation of IκBα, attenuating the NFκB signalling [117]. Luteonin is a plant flavonoid with antioxidant and immunomodulatory properties. A study shows that pre-treatment with luteonin stabilizes Sarco/endoplasmic reticulum Ca^2+^-ATPase (SERCA2a) by stabilizing SUMO1 protein binding and aids in the SUMOylation of SERCA2a specifically at K585 by SUMO1 [41]. SUMO conjugated SERCA2a enhances the mitochondrial membrane potential, rescuing the cell from ischemia reperfusion injury mediated apoptosis [21, 41, 54]. A group of investigators showed that myocardial ischemia reperfusion injury activates DJ1- an E3 ligase which inhibits the action of SUMO1 modified Drp1 [102]. In the absence of this event, DRP1 would be recruited to the mitochondria, activate mitochondrial fission followed by release of caspase 3, promoting infarction expansion [102]. A study with an adeno-associated vector type 1 gene transfer of SUMO1 in swine models with ischemic heart failure showed an improvement in left ventricular ejection fraction and a decrease in left ventricular volume compared to saline treated controls [107].

#### Congenital heart defects

Gam1, an adenovirus protein, selectively leads to the degradation of SUMO E1 activating enzyme [11]. Microinjections of Gam1 mRNA in embryos at different developmental stages revealed sensitivity of non-canonical Wnt/PCP, Ets-1 and snail/twist to the loss of SUMOylation [8]. This loss of SUMOylation further manifested in defects of neural tube formation and heart development [8]. Importantly, Wang et al. demonstrated atrial and ventricular septal defects leading to premature death of SUMO1-deficient mice [109]. Furthermore, to substantiate these findings in humans, they reported a single 16 bp substitution in the regulatory sequence of SUMO1 gene sequence, probably causing loss of SUMOylation activity, in newborns with atrial septal defects and cleft lip [109].

A single point mutation in any transcription factor can largely disrupt the developmental orchestration, ultimately leading to congenital defects. Nkx2.5 SUMOylation deficient mutant added with haplosufficiency led to congenital heart defects in mouse hearts, indicating the importance of SUMOylation in development [57]. An increasing number of mutations in regulatory transcription factors like GATA, Myocardin, Mef2c, Nfatc1, tbx5, SRF, Smad4 have also been discovered which form the basis of numerous congenital defects [17, 43, 44, 69, 78, 82, 93]. Interestingly, these proteins have also been suggested to be modulated by SUMOylation being SUMO substrates [4, 5, 28, 71, 73, 79, 88, 110–112]. However, the relation between these mutations and altered SUMO conjugation is yet to be established.

#### Ion channel defects and cardiac arrhythmias

Ion channels are the key regulators of electrical conduction system of the heart, and impaired function typically causes cardiac arrhythmias. The channels producing the slowly activating K+ currents, called the IKs channels are crucial for ventricular repolarization. A recent study shows that all the four KCNQ1 subunits of the IKs can potentially be SUMOylated and addition of each SUMO molecule shifts the half maximum activation voltage to the positive side [118]. It is known that Kv1.5 mediates the ultra-rapid delayed rectifier potassium current in cardiomyocytes. Studies show that Kv1.5 are also SUMO conjugatable, and that SUMOylation of Kv1.5 can modulate the excitability of cardiomyocytes [6]. SUMO also works as the “gatekeeper” of K2P1 channels, channels that help maintaining the resting membrane potential of the cells [92]. It was shown in oocytes that non SUMOylable K274E and K274R mutant K2P1 channels were constitutively active while the SUMO modified wildtype is silent [92]. To further understand this phenomenon, another group tried to study both the mutations and how these mutations differ from each other [24]. To their surprise, K274E showed an increased current density in K21P1 but K274R failed to show the upsurge, suggesting the increment is actually because of the glutamate and not SUMO dependent in COS7 cells [24]. To further add insights to this notion, Kim and Kang induced oxidative stress to impel deSUMOylation in rat cardiomyocytes using buthionine sulfoximine and hydrogen peroxide, but failed to see any activation in the K+ channels [55]. This puts the role of SUMO in K2P1 regulation in question and demands in-depth research for answering the same. Transient receptor potential cation channel subfamily M member 4 gene (TRPM4) encodes a protein highly expressed in the purkinje fibres, which is a non-selective calcium activated ion channel which depolarizes the membrane by mediating the transport of monovalent cations [61]. A mutation from G to A transcribes a defective protein TRPM4 E7K which is constitutively SUMOylated, insensitive to deSUMOylation [61]. This constitutively SUMOylated mutant does not get endocytosed and remains on the cell membrane, thus impairing the action potential and causing arrhythmias [61].

## Clinical and translational relevance of SUMO

As SUMOylation is intimately involved in various signalling pathways relevant for cardiovascular disease, an obvious question is how this knowledge could be exploited for translation to new diagnostics and therapies. Years of research have produced a plethora of molecules which can inhibit the entire SUMOylation cascade just by inhibiting one of the players [15, 125], for instance, by inhibition of SUMO E1 activating enzymes. Molecules inhibiting the E1 enzyme mostly act as AMP mimics and bind to the E1 enzymes instead of AMP hence further hampering the SUMO-E1 attachment [104]. These compounds are investigated for treating various cardiovascular, neurological, inflammatory, and proliferative disorders [104]. Ginkgolic acid is a well-known compound that inhibits the SUMO E1 enzyme via the same mechanism [91]. Studies show that delivery of Ginkgolic acid via osmotic pumps embedded in the back of MI mice reduced MI induced cardiac fibrosis [91]. Inhibition of E1 enzyme limited both the SUMOylation of PML, hampering the TGFβ1 pathway as well as angiotensin II induced fibroblast to myofibroblast transformation [91]. Inhibitors of SUMO E2 enzyme like Spectomycin B1, directly bind to Ubc9 and inhibit its SUMO binding [38]. Treating the MCF7 breast cancer line with Spectomycin B1 restrains estrogen dependent proliferation of these cells [38]. Hemopoietic lineage switch 5, a RING finger B-box protein with a coiled coil domain, get SUMOylated by SUMO1 [114, 125]. Interestingly, HLS5 can also bind to both Ubc9 and PIAS1 and degrades the SUMO E2 and E3 via its coiled coil domain. SENP inhibitors generally bind to the catalytic site of SENPs impeding their action [125]. These inhibitors can be used to reduce viral infectivity and are also candidates for anticancer therapy [125]. Poly-SUMO chain inhibitors normally have two components, a metal nanoparticle and a modified SIM mimic conjugated to each other via a thiol tail [66]. These compounds get attached to SAE and then get transferred to Ubc9 and eventually to the SUMO substrate, restricting the entry of SUMO molecules in the cascade [125]. Since SUMO 2/3 also contain SUMO binding sites, the metal nanoparticles attach to SUMO 2/3, hampering the poly-SUMOylation process [125].

Another example is sumoylation of SERCA2a, which has long been in the focus of investigators working on heart failure, since it regulates the calcium fluxes in cardiomyocytes necessary for proper contractile function. In 2015, the compound “N106” was identified which could increase SUMOylation of SERCA2a in a heart failure mouse model. N106 increases SUMOylation by activating SUMO E1 enzymes, thereby increasing SERCA2a activity and subsequently contractile function. But targeting a specific SUMO E3 enzyme could turn out to be more definitive as SUMO E1s fail to convey specific effects.

Cardiomyocyte metabolism is tightly regulated, as a slight dysfunction in the energy balance could be catastrophic for a cell type with such a high energy consumption. AMP activated protein kinase (AMPK) is an enzyme which plays a pivotal role in the metabolic homeostasis of a cell with the AMPK pathway shown to be associated with cardiovascular diseases as well [3]. AMPK is activated via interaction with the upstream molecule liver kinase B1 (LKB1), cardiomyocyte-specific deletion of which leads impaired AMPK activation and cardiac dysfunction [46]. Interestingly, during an energy crisis, LKB1 gets sumoylated by SUMO1 at lysine 178, thus enabling its interaction with AMPK [94]. Site-directed mutagenesis studies have demonstrated that the SUMOylation-deficient LKB1 resulted in defective AMPK signaling and malformed mitochondrial function, inducing death in energy-deprived cells [94]. Targeting LKB1 sumoylation could thus be an effective measure against heart diseases. Protection against ischemia is also a common physiological phenomenon in ground squirrels during the state of torpor. Interestingly, the reduction in blood flow to the brain in this context is accompanied with a drastic increase in Ubc9 expression along with a spike in global SUMOylation in the brain and other organs, playing a cytoprotective role. In vitro studies also show a similar response of cells upon oxygen/nutrient deprivation. In conjunction to this, Lee et al. used a flavonoid Quercetin which inhibits SENPs and rescued neuronal cells against ischemia in vitro. It would be fascinating to know if Quercetin could serve the same protective role in the heart against ischemic injury.

Another study suggested the presence of giant fibrillar centres (GFC), large stores of ribosomal gene transcriptional machinery components, in rat sensory ganglia neurons, and reported that the number of GFCs increased with the increase in the cell size [13]. The point of interest here is that they also observed an upsurge of Ubc9 and SUMO1 with GFCs [13]. This data along with other studies suggest a direct role of SUMO in cellular growth. Further investigations regarding targeting SUMO as a therapeutic measure against cardiomyocyte hypertrophy are therefore necessary.

Alterations in mitochondrial dynamics have been reported in almost all cardiovascular diseases and DRP1 thus seems to be an attractive candidate for therapy. After a successful pilot study, a clinical trial on inhibition of the mitochondrial fission protein, drp1, for the prevention of atherosclerosis is under way [ClinicalTrials.gov Identifier: NCT03980548]. Recently, SUMO entered the world of clinical trials when an inhibitor of SUMO conjugation cycle TAK-981 was tested in patients with neoplasms or lymphomas in a phase 1 clinical trial [ClinicalTrials.gov Identifier: NCT03648372]. TAK-981 is derived from ML-792, and both inhibit the SUMO cycle by binding to the C terminal of the SUMO molecule, rendering SUMO E1 impotent to transfer the SUMO protein to E2.

An important point to emphasize is the necessity to find the difference between the exact roles of each of the SUMO molecules along with the upstream regulators of the respective SUMO pathway to impartially target any pathophysiological condition. Another important aspect which needs further clarification are the mechanisms involved in substrate specificity as well as SUMO isoform preference. Also, as we have seen earlier, on how the SUMOylation–ubiquitination axis and the SUMOylation–phosphorylation axis decides the fate of their substrate, unearthing more such interplays would definitely provide a better understanding.

## Conclusions

So, is SUMO ‘*a friend or a foe’*? It would be too early to comment as SUMOylation studies still have a lot to uncover. As of now, it looks like SUMO has context-dependent roles, so SUMO can either be a friend or a foe. The SUMO machinery could definitely be pertinent in understanding the mechanics of cardiovascular diseases as well as finding plausible targets for therapy.

## Data Availability

Not applicable.
